# Migrant Farmworkers' Acceptability of Health Services in Spain: Barriers and Facilitators Identified by Professionals

**DOI:** 10.1111/hex.70147

**Published:** 2025-01-09

**Authors:** Lena van Selm, Iratxe Pérez‐Urdiales, Miquel Úbeda‐Pavia, José Tomás‐Mateos, Maria del Mar Jiménez‐Lasserrotte, María del Mar Pastor‐Bravo, Ana Requena‐Méndez, Erica Briones‐Vozmediano

**Affiliations:** ^1^ ISGlobal Barcelona Spain; ^2^ Faculty of Medicine and Health Sciences University of Barcelona (UB) Barcelona Spain; ^3^ Department of Nursing I University of the Basque Country (UPV/EHU) Biscay Spain; ^4^ Biocruces Bizkaia Health Research Institute Biscay Spain; ^5^ Department and Faculty of Nursing and Physiotherapy University of Lleida Lleida Spain; ^6^ Consolidated Research Group in Society, Health, Education and Culture (GESEC) University of Lleida Lleida Spain; ^7^ Research Group in Healthcare (GRECS) Biomedical Research Institute (IRB) Lleida Spain; ^8^ Department of Nursing, Physiotherapy, and Medicine University of Almería Almería Spain; ^9^ Department of Nursing University of Murcia Cartagena (Murcia) Spain; ^10^ Department of Medicine Solna Karolinska Institute Stockholm Sweden; ^11^ Biomedical Research Networking Center (CIBER) of Infectious Diseases Carlos III Health Institute (CIBERINFEC, ISCIII) Madrid Spain

**Keywords:** agricultural workers' diseases, farmers, health services accessibility, qualitative research, Spain, transients and migrants

## Abstract

**Background:**

Seasonal migrant farmworkers (SMF) make up a significant part of Spain's agricultural labour force. Due to precarious labour conditions, housing insecurity and factors related to migration, SMF are at risk of specific health issues and occupational accidents. In addition, migrants in Spain face barriers when accessing healthcare services. This study explores factors that influence the acceptability of healthcare services among SMF in Spain from the point of view of professionals working with this population.

**Methods:**

Semi‐structured interviews were conducted among 92 professionals working with SMF in four regions of Spain, including NGO workers, healthcare workers, employees of worker unions, public social services and governmental institutions. A thematic content analysis was performed using Atlas.ti.

**Results:**

Professionals identified several barriers and facilitators that influence the acceptability of healthcare services among SMF. The main identified barriers were language, different perceptions of health and healthcare between SMF and Spanish professionals, a limited understanding of the Spanish healthcare system, and precarious working and living conditions. The main identified facilitators were professionals taking time to explain healthcare procedures and rights to SMF and support and information from friends, family and other community members.

**Discussion and Conclusion:**

To overcome barriers, the use of translational services and cultural mediators should be increased. In addition, educational interventions are needed for migrants to better understand the Spanish healthcare system and for healthcare workers to provide culturally appropriate care to migrant patients. Finally, it needs to be considered that inequalities in health and healthcare between SMF and the Spanish native‐born population reach beyond healthcare institutions. They are rooted in structural factors, which include their living and working conditions, social exclusion, and discrimination.

**Patient or Public Contribution:**

As this study, which is part of a bigger project, aimed to focus on healthcare access mainly from the healthcare system perspective, patients and service users were not involved in this part. Another sub‐study within the project will focus on the experience of SMF. Caregivers were included as study participants and despite not having been directly included in the study design, the open‐ended questions used in this study allowed them to bring up the topics they considered important in the context of this study.

## 1 Introduction

Seasonal migrant farmworkers (SMF) make up an essential labour force in the agricultural sector in many European countries. In Spain, in 2023, 197,400 agricultural workers had a foreign or double nationality (25.7% of the total workforce) [1]. However, actual numbers are expected to be much higher as official numbers do not include informal work often performed by undocumented migrants.

In Spain, roughly three groups of SMF with different contract types can be distinguished: SMF with a residence permit and formal contract (indefinite or temporal), circular seasonal migrants recruited under mobility schemes in their country of origin, and undocumented migrants working without a formal work contract [2, 3]. The latter is the most vulnerable group without regulated labour rights. Many live in shantytowns close to the agricultural sites, often without access to running water and electricity [4, 5, 6]. These factors affect their health and well‐being and put them at risk for social exclusion [7, 8].

Upon arrival in Spain, migrants are typically healthier than the general Spanish population, known as the healthy migrant effect. In addition, physically demanding jobs, such as those in agriculture, require physically fit workers [9]. Despite perceiving their health as good [4], working conditions put SMF at risk of specific health issues and occupational accidents. In Europe, the most common health issues reported among SMF are musculoskeletal disorders, dermatological problems, gastrointestinal and respiratory infections, anxiety, stress and depression [8]. In Spain, SMF had 91% more occupational accidents than native farmworkers [10].

### 1.1 Theoretical Framework: Migrant's Accessibility to Health Services

Migrants in Spain face barriers to accessing healthcare services [11, 12], which is influenced by personal and systematic factors. Systematic factors include administrative procedures, geographical closeness and health system capacity [13]. In addition, legislation in Spain has changed from a restrictive regulation between 2012 and 2018 (RDL 16/2012) which did not allow undocumented migrants access to healthcare services in most regions, to universal health coverage from 2018. Although this change made healthcare access more inclusive, it was accompanied by administrative procedures that were sometimes unclear for both migrants and employees of healthcare services, resulting in different implementation between regions and confusion about undocumented migrants' rights that could limit access [11]. Systematic barriers identified for SMF specifically include not being able to get a healthcare card due to lack of identity document or a municipality registration, being unable to visit healthcare centres within their opening hours due to long working days, and healthcare centres being far away from the agricultural sites [5, 11, 14].

Personal factors affecting access to health services include culture, religion and language. Social structures and contexts define how people perceive health, well‐being and healthcare [15, 16]. Therefore, culture plays a fundamental role in how health is experienced and how health‐related services are used and accepted [16, 17]. Two conceptual frameworks explain the concept of acceptability of healthcare services in a complementary manner. Tanahashi (1978) defined it as the willingness of people to use a service, which is related to religious, financial and other personal factors [13]. Levesque et al. described the concept as the possibility of people accepting (aspects of) the services based on cultural and social factors [18]. In this study, we explored factors that influence the acceptability of healthcare services among SMF in Spain from the point of view of professionals working with this population.

## 2 Methods

This paper describes a sub‐study of a broader multi‐method study which focuses on the living and working conditions of SMF and how this impacts their health.

This sub‐study used a descriptive qualitative design in which semi‐structured interviews were conducted. Data collection occurred from January to October 2021 in four Spanish autonomous regions: two in the south, Andalusia (Huelva and Almería provinces) and Murcia, and two in the north, Catalonia (Lleida province) and La Rioja. These areas were selected because they include Spain's highest concentration of agricultural sites and SMF. The researchers used an intentional sampling approach, selecting participants in healthcare services, social services, nongovernmental organizations (NGOs), regional governments, or labour unions based on their experience working with SMF. Snowball techniques were used to obtain additional participants. The inclusion criterion was having worked with SMF for at least 1 year. Finally, 95 professionals were invited to participate, of which 92 participated (Table 1)—three people were unable to participate due to work commitments.

**Table 1:** Sociodemographic characteristics of the study participants (*n* = 92).

	Autonomous region				
	Andalucía	Catalonia	La Rioja	Murcia	Total
Sex					
Female	25	10	13	7	55
Male	6	14	7	10	37
Sector of employment					
Healthcare^a^	9	9	3	2	23
Public Social services	1	0	2	0	3
NGOs	21	11	5	6	43
Worker Unions	0	2	6	9	17
Governmental institutions^b^	0	3	2	1	6

^a^ Including physicians, nurses, social workers, midwives, administrative employees, psychologists, cultural mediators and COVID trackers.

^b^ Members of the regional council and employees of the city council.

### 2.1 Data Collection

After explaining the protocol, participants provided verbal and written informed consent. Due to the COVID‐19 pandemic, some interviews were performed online, all lasting 45–90 min. A script with open questions was used based on five thematic blocks: services provided to SMF, migratory journey, living conditions, health and access to health services, and needs for improvement.

### 2.2 Data Analysis

The interviews were transcribed verbatim in Spanish and imported into the ATLAS.ti web software. We conducted a thematic content analysis to explore barriers and facilitators to the acceptability of healthcare services among SMF. First, we used a deductive approach to code the data, categorizing them as either barriers or facilitators. Subsequently, we adopted an inductive approach to generate additional codes, allowing themes to emerge from the data organically. We then reviewed and refined these codes to develop overarching themes. The thematic analysis was conducted in several phases, as outlined by Braun and Clarke [19]. We conducted and reported the study using the Consolidated Criteria for Reporting Qualitative Research (COREQ) [20]. The participants' quotes included in this paper were translated by the co‐authors and then revised by a bilingual native English speaker.

### 2.3 Ethical Statement

This study followed the principles of the Declaration of Helsinki and the Belmont Report. The project obtained ethical approval from the Arnau de Vilanova University Hospital ethics committee (Lleida, Spain) with code CEIC‐2459.

Before data collection, the researchers provided oral and written information about the project and participants had the opportunity to raise any concerns or questions about the research. All study participants provided written informed consent. A number was assigned to each participant to preserve confidentiality.

### 2.4 Rigour

Methodological rigour was ensured throughout the study following the Guba and Lincoln quality criteria [21]. Credibility, dependability, confirmability and transferability were used as quality criteria to guarantee the rigour of the research process. Credibility was enhanced by the triangulation of the interpretation of the analysis results among different researchers and by providing verbatim quotes in the results section to support their joint interpretation. Dependability was guaranteed as five authors collected and codified the data independently. Then, two of the authors made an independent proposal for the results, discussing and agreeing later on among all the authors the most appropriate one. Confirmability was also enhanced as all the authors have lengthy experience in qualitative research and migration and by the heterogeneity of the participant profiles, all with broad experience working with SMF. Transferability was achieved through the description of the context and the participant profiles.

## 3 Results

The 92 participants were primarily female (59.8%) and worked in NGOs (*n* = 43), healthcare centres (*n* = 23), labour unions (*n* = 17), governmental institutions (*n* = 6) and public social services (*n* = 3) (Table 1).

Findings were categorized into barriers and facilitators. The three barriers were as follows: (1) language and communication challenges, (2) cultural and perceptional barriers and (3) socioeconomic and financial barriers. The three facilitators were as follows : (1) overcoming language barriers in healthcare facilities, (2) informing SMF about procedures in the Spanish healthcare system and (3) cultural competence of healthcare professionals (Table 2).

**Table 2:** Groups, themes and subthemes.

Group	Themes	Sub‐themes
Barriers for acceptability of healthcare services	1. Language and communication challenges	1.1. Language barriers and translation needs
		1.2. Lack of systemic understanding and legal awareness
	2. Cultural and perceptional barriers	2.1. Migrant's understanding of health and healthcare according to professionals
		2.2. Focus on acute health issues and loss to follow‐up
	3. Socioeconomic and financial barriers	3.1. Fear of missing work and economic constraints
		3.2. Precarious Living Conditions
Facilitators for acceptability of healthcare services	4. Overcoming language barriers in healthcare facilities	
	5. Informing SMF about their rights and procedures in the Spanish healthcare system	5.1. Information provided by NGOs and healthcare facilities
		5.2. Information sharing within the community
	6. Cultural competence of healthcare professionals	

### 3.1 Barriers for Acceptability of Healthcare Services

#### 3.1.1 Language and Communication Challenges

##### 3.1.1.1 Language Barriers and Translation Needs

Almost all professionals agreed that language was the main barrier to accessing healthcare services. Many SMF did not speak Spanish and could only access healthcare services through someone who translated. Therefore, SMF inability to explain themselves and administrative and healthcare personnel not having time or capabilities to deal with this issue was reported to be a barrier to SMF acceptability and access to healthcare services.

How do you want a doctor to diagnose a case of someone who does not speak Catalan or understand Spanish? And really, medication and treatment, of course it is impossible. It is difficult.

– I32, cultural mediator, Catalonia

In the field of mental healthcare, language was mentioned as a significant barrier that would prevent SMF from receiving proper care and, therefore, could cause relatively small mental health issues to get worse.

Sometimes, family members serve as translators. However, this could cause problems when discussing sensitive information, such as problems between spouses or when children are asked to translate sensitive details.

It is a problem because when you have to talk about social services related to a sensitive family problem, imagine…

– I39, cultural mediator, Andalucía

Language barriers also affect long‐term care and health needs. For example, people do not attend follow‐up appointments because they do not understand that they must.

According to the participants, many migrants, especially women, wanted to learn the Spanish language but faced barriers. For example, they did not have transport to the town for classes, or their husbands did not want their wives to learn Spanish. Furthermore, participants mentioned that SMF, mainly from sub‐Saharan Africa, often had low levels of education and several were analphabetic. However, participants mentioned that SMF who had been in Spain longer were often well‐adapted, usually spoke Spanish, and could attend their medical appointments autonomously.

##### 3.1.1.2 Lack of Systemic Understanding and Legal Awareness

In Spain, everyone, regardless of legal status, can access emergency care, and everyone registered at a Spanish address can apply for a healthcare card to get primary and specialist care for free. In spite of this, participants mentioned that many SMF, especially when undocumented, were not aware of these rights and often thought that they had to pay for healthcare services.

The relationship with the healthcare system in the country of origin conditions how you are going to relate to the healthcare system in Spain. And there [the country of origin], there are many corrupt systems where you have to pay. Here, many think “here I have to pay everything,” and you have to tell them that they have the right to free healthcare.

– I12, project coordinator NGO, Murcia

Participants emphasized the need for SMF to be well‐informed about the functioning of the Spanish healthcare system. SMF often lack the understanding that when they move between regions, their medical history, including tests and prescribed medication, is not easily accessible to the new physician. Moreover, it was noted that some SMF require assistance navigating the healthcare system beyond just reaching the doctor. For instance, some SMF are unaware of how to obtain medication after receiving a prescription from the doctor.

Another barrier that some participants mentioned was that some newly arrived migrants avoided interacting with the healthcare system because they feared legal issues, even when having a healthcare card. However, a healthcare worker in Andalucía noted a positive shift in attitudes, stating that migrants are now less reluctant to show their passports or give their real names compared to before.

#### 3.1.2 Cultural and Perceptional Barriers

##### 3.1.2.1 Migrant's Understanding of Health and Healthcare According to Professionals

SMF typically comes from countries with healthcare systems different from the Spanish one. Participants said some used traditional medicine and had never attended a healthcare service using biomedical medicine before. Therefore, some SMF expressed distrust towards the Spanish health system and preferred traditional practices they were familiar with, like infusions, plants, bandages and oils with garlic.

“This [garlic] replaces the antibiotic.” Some people say that here, I have been told this several times within the Moroccan collective.

– I12, project coordinator NGO, Murcia

Some migrants visited traditional medicine practitioners, for example, for mental health issues, as this is perceived very differently in many of the migrants' home countries compared to Spain. In addition, participants said that migrants who travel from their countries of origin often bring medicines with them. One reason for using traditional medicine was adhering to what they had learned and believed. However, another reason could be not knowing how to explain their complaints to a healthcare professional in Spain. Participants noted that as migrants stayed longer in Spain, the use of traditional medicine practitioners and drugs from their country of origin reduced and many migrants started using conventional medicine as they developed more confidence in the Spanish system and saw that medicines could benefit them. When managing health, some migrants used personal methods, such as herbs, in combination with treatment provided at the hospital.

SMF often had different expectations and understandings of health and healthcare than Spanish healthcare professionals. They sometimes refused to participate in practices in the Spanish system, such as drawing blood for testing or taking medication. Their focus was on receiving immediate treatment to be cured, rather than undergoing ongoing care and returning for follow‐up appointments or tests.

They often argue “Something hurts me, I go to the doctor, and they have to cure me”. If they cannot cure me immediately, then why am I going to finish the treatment because, in 2 days, the symptoms have not gone away?

– I56, agricultural workers union, La Rioja

The perception of the role of a healthcare professional also differed between cultures. For example, participants explained that in many cultures from sub‐Saharan Africa, people perceive a healthcare professional as an authority figure who knows everything without asking questions. Therefore, participants mentioned that some SMF did not understand healthcare professionals asking about symptoms.

Finally, for cultural and religious reasons, many migrant women did not want to be treated by male healthcare professionals, especially in gynaecologist. They would not attend appointments when seeing that the doctor was male. Some male migrants also had problems taking off their clothes in front of a doctor.

##### 3.1.2.2 Focus on Acute Health Issues and Loss of Follow‐Up

Most SMF only interacted with the health system when they had an acute health issue that prevented them from working and rarely came for check‐ups or prevention or follow‐up. This could be explained by being used to healthcare systems that function in this way, not wanting to miss work and lose income, and moving around for work. Therefore, focusing on prevention was challenging even though many long‐term health concerns of SMF were related to their heavy physical work, which could be partially prevented by health education. A participant mentioned often seeing recurring health issues among SMF because of not taking the prescribed treatment correctly.

When they sometimes do not understand that there are health problems and that the treatments could be long, they don't complete the treatment well, which complicates things, right? It extends a lot, and I don't know, it could be a cultural problem, a lack of understanding of how to take the treatment: “Here I need to take a pill, and it needs to cure me,” and no, there are health problems that do not only require one pill to get cured and sometimes other measures are necessary.

– I64, healthcare worker in NGO, Andalucía

On the other hand, an NGO worker explained that some migrants were used to going to the health centre more frequently in their home counties, and they had to explain to them that they should only go when necessary.

#### 3.1.3 Socioeconomic and Financial Barriers

##### 3.1.3.1 Fear of Missing Work and Economic Constraints

Many SMF did not go to the doctor during working hours out of fear of being fired. In addition, they often lived in precarious situations and could not afford to miss a day of salary. Many SMF had irregular working schedules, and if they had the opportunity to work, they would take it, even if they had a healthcare appointment scheduled.

Everything is related to precariousness. If I have a health problem and I go to the doctor, that day I won't be paid, and in addition, in the conditions in which they are paying me, it is hard for me to recover [financially] or pay my rent. Well, I leave the medical visit to see if it passes [the medical issue], and I go the emergency care unit, and there I manage however I can.

– I41, Agricultural Labor Union, Murcia

As a result, participants mentioned that SMF often went to the emergency care unit, even if their complaints were not directly urgent, since this was the only service they could attend outside their working hours. In other cases, SMF who missed an appointment would show up another day instead. While sometimes they would be attended, mostly they would get a new appointment which could be missed again. Finally, not wanting to miss work led SMF to only seek care when not being able to work anymore, with severe complaints that could have often been prevented. Finally, prioritizing work over health could lead to nonadherence to treatment guidelines. An example was provided of an SMF with a medical issue who had to stay home to avoid getting infected but refused to do so.

##### 3.1.3.2 Precarious Living Conditions

Many SMF live in precarious conditions, which limit the effectiveness of healthcare. In La Rioja, it was mentioned that some SMF slept in the streets despite the obligation of employers to provide them with housing for the duration of the contract. Possibly because SMF did not want to stay in the shared rooms provided by their contractors or due to (temporarily) unemployment. Even for SMF who are not homeless, precarious living conditions can hinder access to optimal healthcare and recovery. An example was mentioned of peritoneal dialysis for kidney disease, a less invasive alternative for haemodialyses, which can be performed at home. A healthcare professional mentioned that a lack of space and privacy at home would often not permit this option, forcing them to come to the hospital for each treatment.

### 3.2 Facilitators for Acceptability of Healthcare Services

#### 3.2.1 Overcoming Language Barriers Within Healthcare Facilities

A significant facilitator for overcoming language barriers was having cultural mediators at the medical appointments so migrants could fully express themselves and share their concerns. According to the participants, most healthcare centres in Almería, Andalusia, would have translators or mediators. However, an NGO employee said that despite hearing about translational services at healthcare services, it was unclear how it worked, and the NGO would always translate. In Catalonia and Murcia, some healthcare centres also mentioned having cultural mediators. A midwife shared how a translator helped her to gain patient's trust and facilitate communication.

Some immigrant organizations in Andalusia offer phone translation services for healthcare facilities. Although healthcare workers considered this service useful, especially for translating specific dialects, it was only used when no other options where available (e.g., translation by family, friends or compatriots) since using this service was perceived as time‐consuming.

Overcoming language barriers also depended greatly on the effort of the personnel at the healthcare centre. An administrative worker described doing everything possible to get the message across so that patients leave with their needs met, even though this could take a long time. Similarly, a midwife explained that, together with a translator, she was making a lot of effort to explain everything well to migrant women. Still, it would take her twice as long as with a Spanish patient.

Not everyone can do it [put in extra time and effort]. You need to be passionate about it and it should not be like that. I think it is an inequality. Because you empathize more or less, they [migrant women] get a completed visit or not.

– I16, healthcare professional, Catalonia

Participants suggested increasing the availability of cultural mediators and translators and emphasized the importance of migrants being able to express themselves in their language. Specific recommendations included cultural mediators rotating between centres on set days, using technology for translations, and promoting existing services, like the translation phone service in Andalusia.

#### 3.2.2 Informing SMF About Their Rights and Procedures in the Spanish Healthcare System

##### 3.2.2.1 Information Provided by NGOs and Healthcare Facilities

An employee at a healthcare facility said they first had to take away fears and teach migrants how to interact with the healthcare centre.

So, the first thing has been taking away those fears (…) that we are not going to inform the police, but that it is really for their good and that today in Andalusia, thank God, the healthcare system is universal, and everyone has the right to access it

– I47, administrative worker healthcare center, Andalusia

They did this by visiting the migrant populations and organizing courses. For temporary migrant workers from EU countries (mainly Eastern Europe), awareness raising consisted of explaining the importance of obtaining a European healthcare card and bringing it to work, which allows them healthcare access.

NGOs educated migrants about how the healthcare system in Spain works, for example, through courses on the differences between regional healthcare systems and infographics in regional languages explaining how to download the healthcare application on their phones and make an appointment. Several NGOs accompanied migrants to their appointments when needed. However, an NGO employee emphasized that teaching them how to go independently in the future is also essential. A cultural mediator explained that they informed SMF the importance of primary care to get a good follow‐up.

[Telling them:] You need to go there [the primary healthcare center] because they will do a good follow‐up there. It is not that they will not help you well at the emergency care unit, but the important thing is that primary care can understand your case; they can study it and see the evolution.

– I14, cultural mediator, Catalonia

A healthcare worker mentioned the importance of finding the right time to provide health education to SMF since they were often very stressed when they came in with acute problems not able to process information in that moment.

In the settlements in Andalucía, where many SMF live, NGO employees monitored if people were taking their medication and going to their medical appointments and shared this information with the nearby healthcare centres. Both improving SMF knowledge of the healthcare system and identifying and supporting SMF that do not reach healthcare were mentioned as suggestions for improvement by participants.

##### 3.2.2.2 Information Sharing Within the Community

Support within migrant communities facilitated the acceptance of care. A healthcare worker said that nowadays, the fear of engaging with the healthcare system has reduced, and migrants encourage each other to share information they get at the healthcare centre. A participant mentioned that most SMF visit healthcare services because someone from their community explains how to get there.

#### 3.2.3 Cultural Competence of Healthcare Professionals

A facilitator for acceptability was a basic understanding of the healthcare system in the patient's country of origin by the healthcare professional. In addition, healthcare professionals being open to respecting the use of traditional and Western medicines, as long as they did not harm, was mentioned to help build trust and increase treatment adherence.

So, when they tell me that they are going to their country to cure hepatitis because traditional medicine can do that, well, I won't tell them no, nor will I express myself against traditional medicine. I tell them, okay, try it. Hopefully, it will cure you. Hopefully, it works for you, but do not let that stop you from coming to the consultations when you return so we can follow up with you and everything. But I don't think you should encourage traditional medicine because for specific illnesses, some potentially severe and fatal, for this population, you should not create false expectations.

– I4, healthcare professional, Andalusia

Participants also suggested that healthcare providers educate themselves more on the healthcare systems where most migrants come from to know when additional explanations are needed. For example, services are expensive in their countries of origin, as this might make them hesitant to accept them in Spain.

It can be challenging for healthcare workers to deal with people from different cultures. Healthcare professionals coming from areas where they barely attend migrants were often shocked at first by the language barriers and social and cultural differences. A healthcare professional explained that cultural competence is not part of a regular medical degree. He explained how it took him years of working with various populations, talking with community leaders, and following courses to understand the culture and mentality of different migrant populations.

[Understanding] the fears that this person may have, that what is important for them is work and that health has a lower priority, and that we have to be aware of when, for example, we need to make attending the consultation more flexible.

– I4, healthcare professional, Andalusia

## 4 Discussion

The main identified barriers to acceptability of healthcare services among SMF were language, different perceptions of health and healthcare between SMF and Spanish professionals, and a limited understanding of the Spanish healthcare system by SMF. Facilitators to overcome these barriers included professionals taking time to explain procedures and rights to SMF and support and information from within the community.

Language barriers, cultural differences and a limited understanding of the healthcare system are known barriers among SMF in Europe [8]. In Spain, healthcare workers and migrant patients described language barriers as a significant issue and mentioned a lack of professional translators and cultural mediators [22, 23, 24]. The lack of translation and cultural mediation services in the healthcare system has been associated with health disparities and discrimination in healthcare provision [25, 26]. In addition, the lack of effective communication between healthcare professionals and patients can generate distrust and misunderstandings [27]. For example, in southern Spain, SMF rated healthcare quality as poor, they reported all getting the same treatment without receiving diagnostic tests, which they attributed to the language barrier [28]. Although some participants in the same study were optimistic about the healthcare they received, attributing this to the personal qualities of healthcare workers [28], which was mentioned as a facilitator in our study as well.

This study found that SMF lacks knowledge of using the Spanish healthcare system. Likewise, in southern Spain, 65% of migrants had inadequate or problematic health literacy [29], meaning they lacked the capacity to obtain, process and understand basic health information and services needed to make appropriate health decisions [30]. Therefore, as the participants of this study suggested, solutions should move beyond solving language barriers and educate SMF on using the Spanish healthcare system.

The findings of this study show the importance of cultural competence among healthcare workers. Cultural competence consists of cultural awareness and sensitivity, cultural knowledge and skills to apply this knowledge to clinical care [31]. Collaboration between culturally competent practitioners and cultural mediators can improve communication with migrant patients and improve their health outcomes [32, 33]. In southern Spain, both nurses and migrants described a lack of understanding among nurses of the migrant patients' cultures and religion, and several nurses reported never having had training on this topic [22, 23]. Likewise, in this region, some undocumented SMF reported a lack of cultural awareness from healthcare staff [28]. Currently, in Spain, among 47 universities with a nursing degree, 61% had included some training on transcultural care or cultural diversity [34]. However, in most cases, this was only taught in theoretical sessions, and these courses were mandatory in only 59% of the universities offering them [34]. Therefore, education on cultural competence should be included in the education of healthcare workers more extensively.

The barriers identified in this study go beyond intercultural understanding within the patient‐provider relationship and are closely related to social inequalities and structural vulnerability. The framework of structural vulnerability explains how factors related to inequality, such as type of work, type of residence, legal status, language, ethnicity and gender, produce collective health inequalities [35, 36]. Many SMF lack a regular status and tend to live in precarious living conditions characterized by substandard housing and overcrowding [37]. As seen in the study results, living conditions and not wanting to miss work, reflecting structural vulnerability, limit SMF access to adequate healthcare services [12]. The concept of structural competence provides a framework that recognizes how health outcomes and healthcare delivery are affected by broader structural factors, including the social determinants of health, structural vulnerability, violence, racism and even the definitions of illness and health used in conventional medicine [38].

### 4.1 Implications

To increase the acceptability of healthcare services, it is suggested to work with interpreters and cultural mediators and provide intercultural competence training for healthcare workers. The services of interpreters and cultural mediators could improve communication and the patient‐provider relationship by enhancing intercultural understanding [39]. With an increasingly culturally diverse population, including cultural competence in nursing and medicine education would be beneficial. In addition, it is crucial to implement health interventions that consider social inequalities and structural vulnerability. The structural and intercultural competence framework could be helpful in addressing health disparities among SMF, as it addresses diversity in healthcare systems and analyses the elements contributing to inequality and structural vulnerability [40, 41, 42, 43]. Moreover, by fostering an understanding of how societal structures impact health outcomes, professionals could better address the root causes of health disparities, potentially leading to more tailored and equitable care provision [43]. A more comprehensive approach in the health field must recognize cultural diversity and social structures and inequalities [44].

### 4.2 Strengths and Limitations

The main strength of this study was the broad spectrum of views that was included since we interviewed participants working in healthcare institutions, NGOs, worker unions and governmental institutions, in the six regions covering the main agricultural sites in Spain. To our knowledge, this is the first study to explore professionals' perceptions of the acceptability of health services among agricultural migrant workers, as the first step of a 6‐year national project that will increase the knowledge of improving migrants' access to health services. The study had some limitations. First, the perspectives of SMF themselves were not included in this study. Second, the interviews were conducted in Spanish and although careful attention was given to ensuring accurate translation, minor nuances or meanings may have been lost or altered during the translation process. Third, SMF are a diverse population with varying nationalities, genders, ages and living and working situations. These individual differences were not further explored in this study. However, in the next phase of the overarching AGROMISALUD project [7], both these aspects will be considered, and interviews with SMF will be conducted.

## 5 Conclusions

According to professionals working with SMF, the main factors affecting the acceptability of healthcare among SMF were language, cultural understanding of health and healthcare, and lack of understanding of the Spanish healthcare system. The use of translational services and cultural mediators should be increased to overcome these barriers. In addition, educational interventions are needed for migrants to better understand the Spanish healthcare system and for healthcare workers to provide culturally appropriate care to migrant patients. Finally, it needs to be taken into account that causes for inequalities in health and healthcare between SMF and the Spanish native‐born population reach beyond healthcare institutions. They are rooted in structural factors, which include their living and working conditions, social exclusion and discrimination.

## Author Contributions

**Lena Selm:** writing–original draft, writing–review and editing, formal analysis. **Iratxe Pérez‐Urdiales:** writing–original draft, writing–review and editing, methodology, formal analysis. **Miquel Úbeda‐Pavia:** writing–review and editing. **José Tomás‐Mateos:** conceptualization, writing–review and editing. **Maria del Mar Jiménez‐Lasserrotte:** investigation, writing–review and editing. **María del Mar Pastor‐Bravo:** investigation, writing–review and editing. **Ana Requena‐Méndez:** writing–review and editing, supervision. **Erica Briones‐Vozmediano:** writing–review and editing, supervision, formal analysis, methodology, funding acquisition, investigation, conceptualization.

## Conflicts of Interest

The authors declare no conflicts of interest.

## Data Availability

The data that support the findings of this study are available from the corresponding author upon reasonable request.
